# Supplementary material for: Dynamic interactions of retroviral Gag condensates with nascent viral RNA at transcriptional burst sites: implications for genomic RNA packaging
Source: mBio. 2025 Aug 19;16(9):e01695-25. doi: 10.1128/mbio.01695-25 (PMC12421996; doi:10.1128/mbio.01695-25)
Supplement: Supplemental Material — Tables S1 and S2, Fig. S1 to S4, and captions for Movies S1 to S9. [file mbio.01695-25-s0001.pdf]

Supplemental Material

Supplemental Table 1. Distance of RSV transcriptional burst from the edge of the nucleus defined by DAPI staining

Supplementary Table 1: Burst distance to DAPI Edge										
**Bin Center Distance From DAPI edge (μm)**	**0.00**	**0.1**	**0.2**	**0.3**	**0.4**	**0.5**	**0.6**	**0.7**	**0.8**	**0.9**

Supplemental Table 2. Distance of Gag from edge of the nucleus defined by DAPI staining

Supplementary Table 2: Gag distance to DAPI Edge																			
**Bin Number Distance to DAPI Edge (µm)**	**0.00**	**0.10**	**0.20**	**0.30**	**0.40**	**0.50**	**0.60**	**0.70**	**0.80**	**0.90**	**1.00**	**1.10**	**1.20**	**1.30**	**1.40**	**1.50**	**1.60**	**1.70**	**1.80**
Number of Gag foci	822	132	100	102	46	30	28	12	15	17	9	6	5	0	4	1	1	0	1

Supplemental Figure 1: Co-localization between Gag.L219A and nascent non-viral RNAs.

A) Schematic of the Gag.L219A-CFP NES mutant and pSL-MS2-24x. **B)** QT6 cells expressing a Gag nuclear export mutant (Gag.L219A) were co-transfected with either pSL-MS2-24x, a non-viral construct containing 24 copies of MS2 stem loops or pulse labeled for 10 minutes with 5-fluorouridine (5FU) to label nascent RNAs. (a) pSL-MS2-24x RNA foci (green) co-localized with Gag.L219A (red) at 18±4% ($p<0.0001$) in the nucleus (white outline). (b) 5FU labeled RNA foci (green) co-localized with Gag.L219A foci at 10±4% $p<0.0001$). **C)** Bar graph of percent co-localization of RNA with Gag.L219A (24). 31 cells from 5 replicates were collected for Gag.L219A+ pSL-MS2-24x and 18 cells from 3 replicates were analyzed for Gag.L219A + 5FU-labeled RNA.

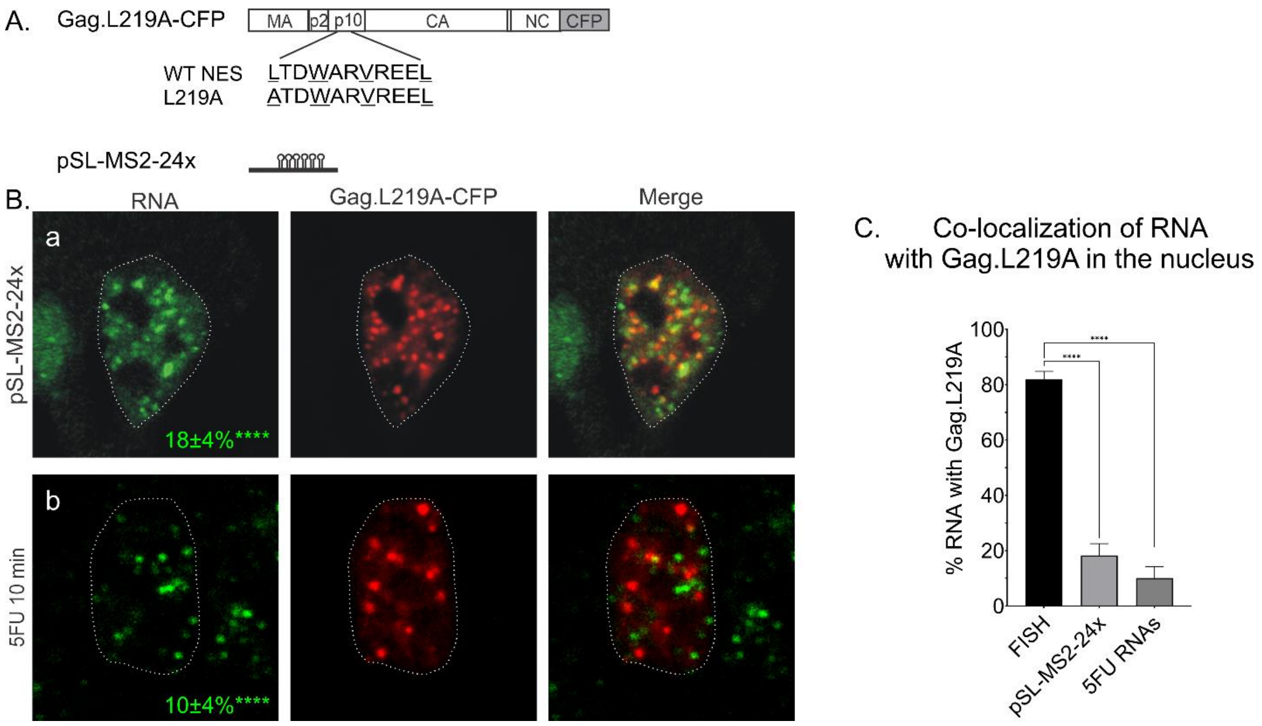

Supplemental Figure 2: STED microscopy of USvRNA bursts reveals complex structures. **A)** Single z-slices of chronically infected cells comparing bursts of transcription imaged via confocal microscopy (red) to those imaged via STED (green). The nucleus is marked with Sun1-venus (blue, white outline). The image below is a zoom in of the burst of interest. The confocal burst appears as a single focus while the STED burst contains multiple smaller foci. Scale bar= 1 µm. **B)** Surface renderings were generated of the cell above and subjected to orthogonal clipping planes at either 0° or 90°. The STED bursts have a more lobed appearance. Scale bar= 1 µm. **C and D)** Two more examples of highly structured USvRNA bursts imaged via STED. The bursts are presented as a single Z-slice (Scale bar= 0.5 µm) or with an X,Y surface slicer (Scale bar=0.3-0.5 µm). In the bottom right corner of the bottom panels, a zoom in of a volume rendering of the bursts are presented (Scale bar= 0.07-0.1 µm). Both bursts appear lobed and highly structured. 3 Replicates.

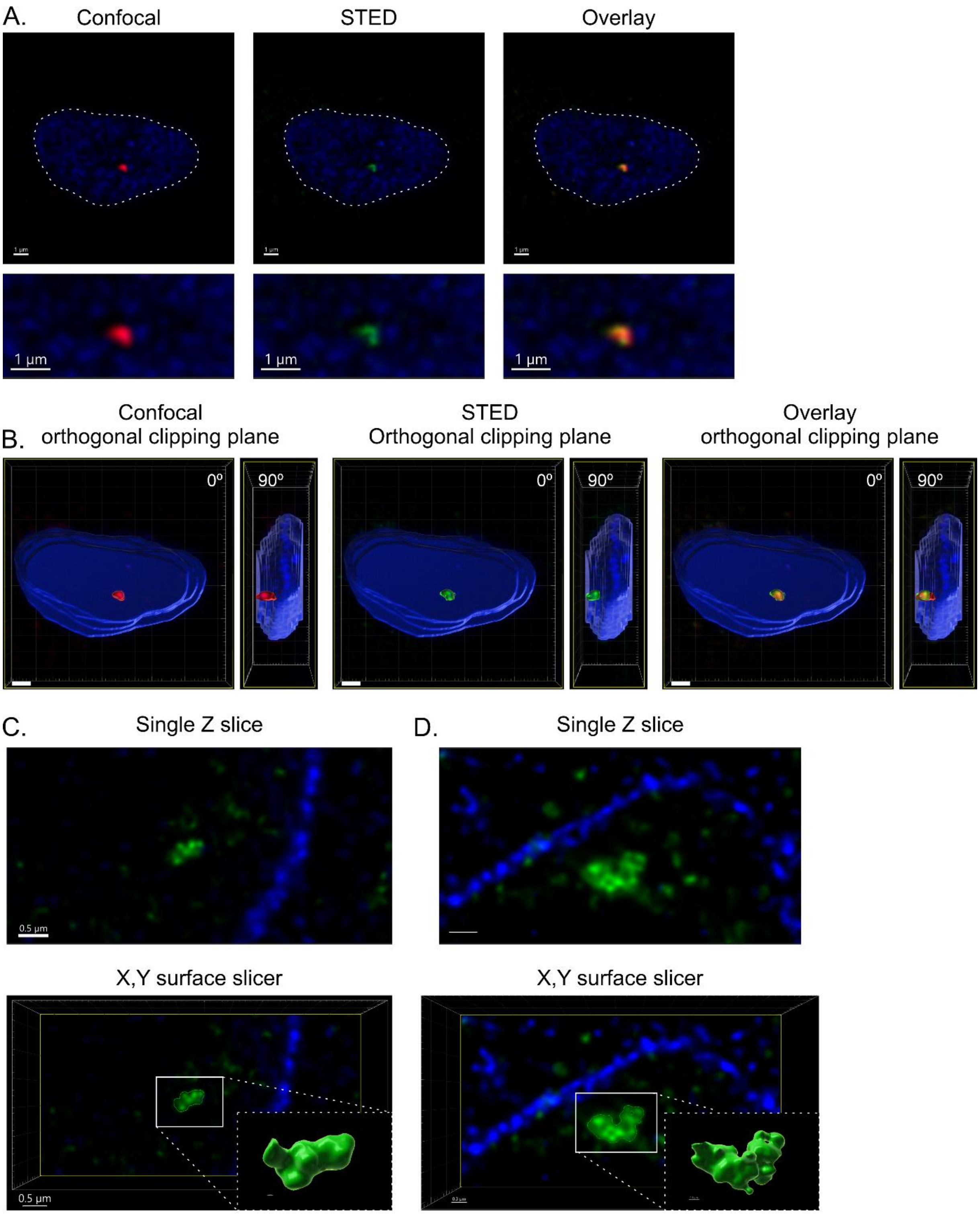
A.
Confocal
STED
Overlay
1 µm
1 µm
1 µm
1 µm
1 µm
1 µm
B.
Confocal
orthogonal clipping plane
STED
Orthogonal clipping plane
Overlay
orthogonal clipping plane
0º
90º
0º
90º
0º
90º
C.
Single Z slice
0.5 µm
X,Y surface slicer
0.5 µm
D.
Single Z slice
X,Y surface slicer

Supplemental Figure 3: Single plasmid controls for BiFC. QT6 cells were transfected with VN-Med26, CTCF-VN, or VN-Med1 alone to confirm that fluorescence did not occur in the absence of Gag-VC. Cells were imaged a low power and adjusted according to VN-Med26+Gag-VC as for the images in figure 10. Scale bar = 10 µm. Six replicates were collected for each condition.

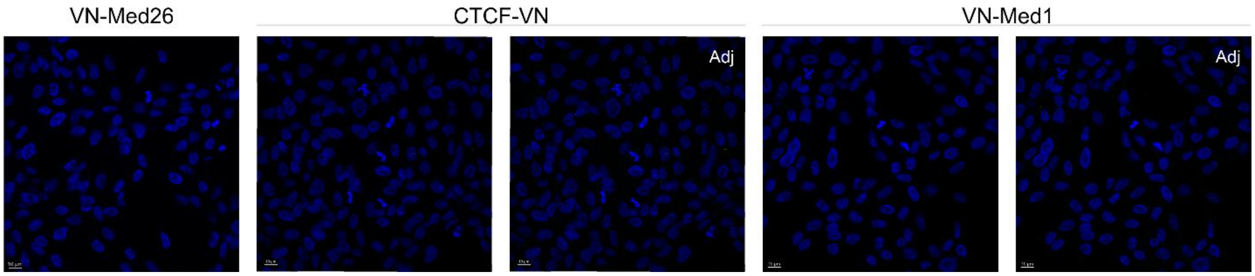

Supplemental Figure 4: Fusion of VN-Med26:Gag-VC BMCs over time. Single Z slices were obtained from stills extracted from Supplementary Movie 9. Fusion of foci is a characteristic of BMCs. **A)** An example of two VN-Med26:Gag-VC foci (white arrows, inset) fusing over a period of 20 minutes. **B)** Another example of two VN-Med26:Gag-VC foci (white arrows, inset) fusing over a period of 10 minutes. Scale bar = 1 µm. 2 replicates.

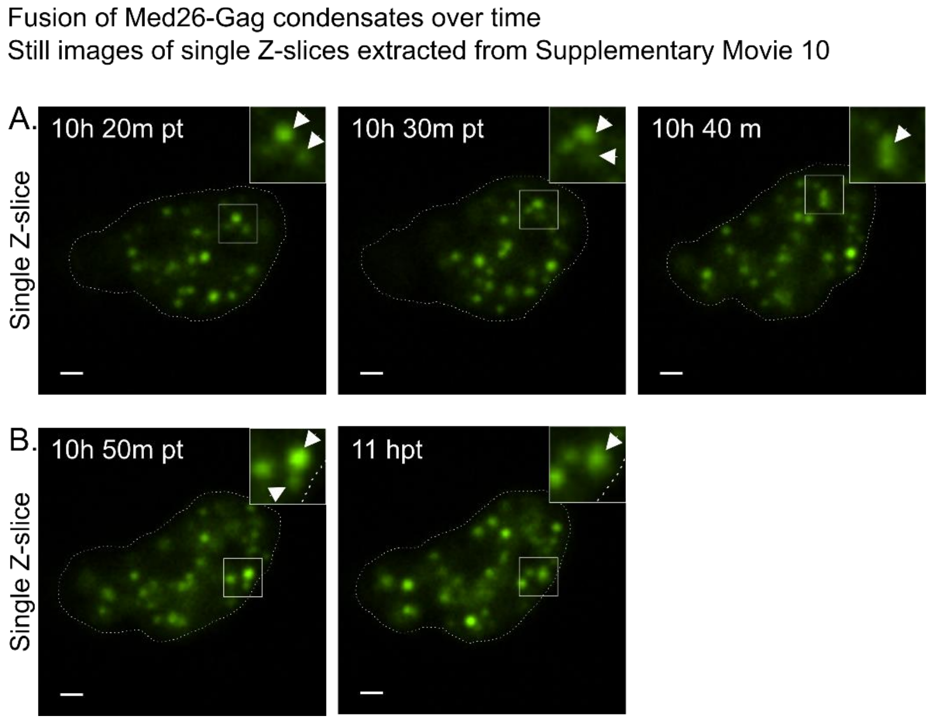

Supplemental Movie Legends:

Supplemental Movie 1: Dynamic movements of Gag and USvRNA foci with particle tracking. The QT6 rtTA TRE RC.V8 MS2 stbl cell line that was transfected with Gag-SNAPTag JF549 (red) and NES1-YFP-MS2-NLS (USvRNA-green) and dox induced for approximately 22 hours. Cells were imaged every second. Particle tracking was conducted using the Imaris spot function. The USvRNA burst (green) and Gag focus (red) transiently co-localized in the nucleus. The nucleus is marked due to the NES1-YFP-MS2-NLS being able to clear the nucleus.

Supplemental Movie 2: Dynamic movement of Gag and USvRNA foci with colocalization channel. This movie shows the overlay of the co-localization channel generated from the USvRNA and Gag signals from Movie 1.

Supplemental Movie 3: Particle tracking showing transient interaction of Gag and USvRNA foci. The QT6 rtTA TRE RC.V8 MS2 stbl cell line was transfected with Gag-SNAPTag JF549 (red) and NES1-YFP-MS2-NLS (USvRNA-green) and dox induced for ~16 hours. Cells were imaged every second. Particle tracking was conducted using the Imaris spot function. A burst of USvRNA (green) in the nucleus was met by a red focus of Gag to undergo a transient interaction. The nucleus was marked based on the NES1-YFP-MS2-NLS signal.

Supplemental Movie 4: Particle tracking of multiple dynamic Gag foci at USvRNA burst. The QT6 rtTA TRE RC.V8 MS2 stbl cell line that was transfected with Gag-SNAPTag JF549 (red) and NES1-YFP-MS2-NLS (USvRNA-green) and dox induced for ~16 hours. Cells were imaged every second. Particle tracking was conducted using the Imaris spot function. Two Gag foci (red) were tracked to the same burst of USvRNA (green). Gag condensate 1: Yellow track. Gag condensate 2: White track. The nucleus is marked based on the NES1-YFP-MS2-NLS signal. This is the same cell imaged in Supplemental Movie 3 but at an earlier time point.

Supplemental Movie 5: Movement of Gag focus into nucleus and colocalization with USvRNA burst. The QT6 rtTA TRE RC.V8 MS2 stbl cell line that was transfected with Gag-SNAPTag JF549 (red) and NES1-YFP-MS2-NLS (USvRNA-green) and dox induced for 2 hours. Cells were imaged every second. Particle tracking was conducted using the Imaris spot function. A focus of Gag (red) was tracked from the cytoplasm into the nucleus and kissed the USvRNA burst (green). The nucleus is marked due to the NES1-YFP-MS2-NLS being able to clear the nucleus.

Supplemental Movie 6: Movement of colocalized Gag-USvRNA focus into the cytoplasm with particle tracking. The QT6 rtTA TRE RC.V8 MS2 stbl cell line was transfected with Gag-SNAPTag JF549 (red) and NES1-YFP-MS2-NLS (USvRNA-green) and dox induced for ~22 hours. Cells were imaged every second. Particle tracking was conducted using the Imaris spot function. A focus USvRNA (green), not correlating to a burst, in the nucleus formed a vRNP with Gag that trafficked from the nucleus into the cytoplasm. The nucleus was labeled with NucSpot 650.

Supplemental Movie 7: Colocalization channel showing Gag with USvRNA burst in cells expressing Nup98 transdominant mutant. The QT6 rtTA TRE RC.V8 MS2 stbl cell line that was transfected with Gag-SNAPTag JF549 (red), NES1-YFP-MS2-NLS (USvRNA-green), and NP98 (to trap Gag in the nucleus), and dox induced for 2 hours. Cells were imaged every second. Particle tracking was conducted using the Imaris spot function. A Gag condensate (red) kissed the USvRNA burst (green). The nucleus is marked via DRAQ5. A co-localization channel (white) was overlaid with the Gag and USvRNA signals to indicate when the condensates “kiss” over time.

Supplemental Movie 8: Colocalization of multiple Gag foci at USvRNA transcriptional burst in cells expressing Nup214 transdominant mutant. The QT6 rtTA TRE RC.V8 MS2 stbl cell line that was transfected with Gag-SNAPTag JF549 (red), NES1-YFP-MS2-NLS

(USvRNA-green), and NP214 (to trap Gag in the nucleus), and dox induced for 2 hours. Cells were imaged every second. Three Gag condensates (red) kissed the USvRNA burst (green). The nucleus is marked with DRAQ5. A co-localization channel (white) was overlaid with the Gag and USvRNA signals to indicate when the condensates “kiss” over time.

Supplemental Movie 9: Live cell imaging of Gag-Med26 BIFC nuclear foci. QT6 cells were transfected with VN-Med26 and Gag-VC. Fluorescence is only visible if Med26 and Gag come into close enough proximity to reconstitute the VN and VC halves into a full Venus protein. Cells were imaged starting at 8 hours post transfection and imaged every 10 minutes for 3 hours. Med26-Gag complexes form over time indicated by the increasing fluorescence and foci formation.
